# Supplementary material for: Rapid adiabatic couplers with arbitrary split ratios for broadband DWDM interleaver application
Source: Nanophotonics. 2025 Sep 1;14(18):3089–94. doi: 10.1515/nanoph-2025-0288 (PMC12442355; doi:10.1515/nanoph-2025-0288)
Supplement: Supplementary file 1 — Supplementary Material Details [file j_nanoph-2025-0288_suppl_001.pdf]

Rapid adiabatic couplers with arbitrary split ratios for broadband DWDM interleaver application

Supplementary Information

1 Optimized parameters for rapid adiabatic couplers

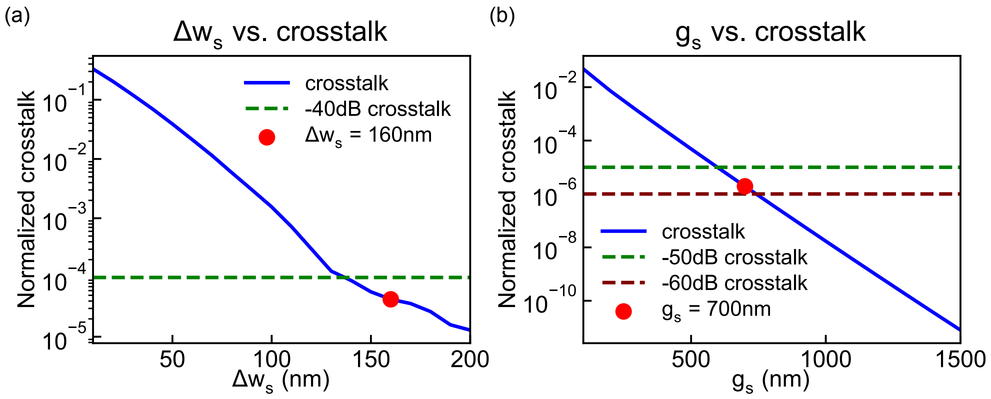

Fig. S.1: (a) Simulated crosstalk as a function of waveguide width difference. (b) Simulated crosstalk as a function of the initial gap between 580 nm- and 420 nm-wide waveguides.

The optimization of the width difference Δw_s and the starting gap g_s proceeded as follows. To balance adiabaticity against propagation loss, we first optimized the width difference Δw_s at the end of Section I. Two S-bend waveguides of unequal width were modeled in a 3D FDTD simulation; the widths were varied symmetrically about 500 nm until the cross-port power at the S-bend exit fell below -40 dB. The resulting curve, plotted in Fig. S.1(a), shows a threshold around $\Delta w_s \approx 140$ nm. Allowing a small engineering margin, we adopted $\Delta w_s = 160$ nm, corresponding to the 580 nm / 420 nm pair used throughout the design.

With the 580 nm / 420 nm pair fixed, an EME sweep was then performed on the initial gap g_s. In each run, the TE_0 mode was launched into the narrower guide, and the TE_0 power emerging from the opposite guide was recorded. The peak value of the resulting oscillatory crosstalk curve was taken as the worst case for that gap. Fig. S.1(b) shows that the worst-case crosstalk first falls below -50 dB at around 600 nm; to provide a safety margin, we chose $g_s = 700$ nm as the starting gap for Section I.

Tab. S.1: Optimized Δw_e values for target split ratios

Target Split Ratio	Δw_e (nm)
71:29	4.84
80:20	10.72
92:8	22.36
96:4	30.30
99:1	42.76

Tab. S.1 lists the optimized values of $\Delta w_e = w_{e,1} - w_{e,2}$ for each target split ratio. The two output waveguide widths were defined as $w_{e,1} = 500$ nm $+ \Delta w_e/2$ and $w_{e,2} = 500$ nm $- \Delta w_e/2$. All values were optimized through parameter sweeps under a 220-nm-thick silicon photonic platform to achieve the desired power splitting at the end of Section II.

Tab. S.2: Total lengths of RACs for each split ratio

Target Split Ratio	Total Length (µm)
50:50	79.9
71:29	115.814
80:20	81.85
92:8	68.042
96:4	64.758
99:1	57.974

Tab. S.2 summarizes the total lengths of the RACs optimized for each target split ratio. These lengths include all three sections of the coupler: Sections I, II, and III.

2 Method for scaling segment lengths

As stated in the main text, we scale the length of each segment in proportion to the local coupling strength for further optimization. Because the local power coupling $|\kappa|^2$ rises almost exponentially–as two waveguides approach in Section I and as the width difference between them becomes smaller in Section II–a simple linear rule $|\kappa|^2 L$ = constant would force very abrupt curvature, which increases bending loss. Instead, we first compress the dynamic range with a logarithmic map and optional flattening,

$$\text{weight}_i \;=\; \big[\log\!\big(1+\alpha|\kappa_i|^2\big)\big]^{\beta},\ \text{scaling\_factor}_i \;=\; \frac{\text{weight}_i}{\sum_j \text{weight}_j}, \tag{S1}$$

and then distribute the total section length according to

$$L_i \;=\; \text{scaling\_factor}_i * L_{\text{section}}, \tag{S2}$$

where L_i is segment length and L_{section} is total section length.

The constants α and β are chosen so that the minimum radius of curvature never falls below 20 µm for 220 nm-thick silicon waveguides. These parameters can be adjusted if a different geometry or material platform requires other optimized translational offset values.

3 Phase delay arm lengths in DWDM interleaver

Tab. S.3: Phase delay arm lengths of each AMZI

AMZI Type	Stage	Length (µm)
Third-order	ΔL_1	361.770
	ΔL_2	710.369
	ΔL_3	712.005
Second-order A	ΔL_1	184.151
	ΔL_2	353.161
Second-order B	ΔL_1	183.676
	ΔL_2	352.211
First-order A	ΔL_1	92.821
First-order B	ΔL_1	92.980
First-order C	ΔL_1	92.901
First-order D	ΔL_1	93.059

Tab. S.3 summarizes the phase delay arm lengths used in each AMZI stage, following the naming convention introduced in Fig. 3(a) of the main text. All values were optimized using the conventional transfer matrix method to achieve the desired spectral characteristics and ITU-grid alignment.

4 Error analysis

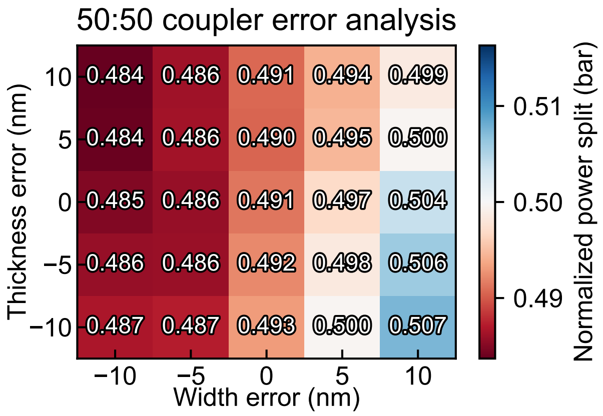

Fig. S.2: Simulated power-split(bar port) variation of the 50 : 50 RAC at 1550 nm under combined fabrication errors.

Fig. S.2 shows the normalized bar-port power, simulated at 1550 nm wavelength, when the waveguide width and Si-film thickness are independently perturbed from -10 nm to +10 nm in 5 nm steps, while all translational offsets remain fixed. Across this full window, the split ratio varies between 0.484 and 0.507, confirming the coupler's tolerance to typical process errors.

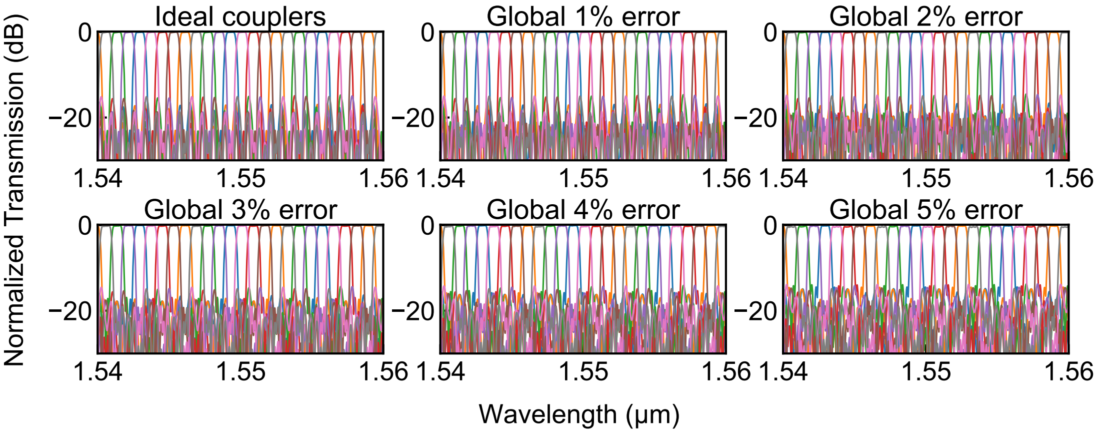

Fig. S.3: Simulated spectra of the 8-channel DWDM interleaver with a uniform splitting-ratio error applied to all couplers.

To quantify the sensitivity of the DWDM interleaver to power splitting ratio errors, we introduced a global deviation(ϵ) into the nominal splitting ratios of all couplers and re-evaluated the eight-channel transfer-matrix model. The calculation assumes loss-free, wavelength-independent couplers; ϵ was swept from 0 % to 5 % while all other parameters were held ideal. The resulting transmission spectra are plotted in Fig.S.3.

The worst-case channel-center crosstalk(bar port) in the central pass-bands degrades from −25 dB for ϵ = 0 % to −22, −19, −17, −15, and −14 dB for ϵ = 1, 2, 3, 4, and 5 %, respectively. Because this sweep ignores the wavelength dependence of the splitting ratio and of the propagation constant, the values represent a best-case limit; including realistic dispersion typically worsens the crosstalk by several decibels.
